# Perspectives of Black, Latinx, Indigenous, and Asian Communities on Health Data Use and AI: Cross-Sectional Survey Study

**DOI:** 10.2196/50708

**Published:** 2025-02-21

**Authors:** Fatuma-Ayaan Rinderknecht, Vivian B Yang, Mekaleya Tilahun, Jenna C Lester

**Affiliations:** 1 San Francisco School of Medicine University of California San Francisco, CA United States; 2 Department of Dermatology University of California, San Francisco San Francisco, CA United States

**Keywords:** augmented intelligence, artificial intelligence, health equity, dermatology, Black, Latinx, Indigenous, Asian, racial and ethnic minority communities, AI, health care, health data, survey, racism, large language model, LLM, diversity

## Abstract

Despite excitement around artificial intelligence (AI)–based tools in health care, there is work to be done before they can be equitably deployed. The absence of diverse patient voices in discussions on AI is a pressing matter, and current studies have been limited in diversity. Our study inquired about the perspectives of racial and ethnic minority patients on the use of their health data in AI, by conducting a cross-sectional survey among 230 participants who were at least 18 years of age and identified as Black, Latinx, Indigenous, or Asian. While familiarity with AI was high, a smaller proportion of participants understood how AI can be used in health care (152/199, 76.4%), and an even smaller proportion understood how AI can be applied to dermatology (133/199, 66.8%). Overall, 69.8% (139/199) of participants agreed that they trusted the health care system to treat their medical information with respect; however, this varied significantly by income (P=.045). Only 64.3% (128/199) of participants felt comfortable with their medical data being used to build AI tools, and 83.4% (166/199) believed they should be compensated if their data are used to develop AI. To our knowledge, this is the first study focused on understanding opinions about health data use for AI among racial and ethnic minority individuals, as similar studies have had limited diversity. It is important to capture the opinions of diverse groups because the inclusion of their data is essential for building equitable AI tools; however, historical harms have made inclusion challenging.

## Introduction

Despite excitement around artificial intelligence (AI)–based tools in health care, there is work to be done before they can be safely deployed. Addressing dataset diversity and racism perpetuated by large language models is paramount [1-3]. The absence of diverse patient voices in AI discussions is a pressing matter, and current studies have been limited in diversity [4,5]. Our study examined the perspectives of racial and ethnic minority patients on the use of health data in AI.

## Methods

### Overview

A cross-sectional survey was administered via Qualtrics to participants aged 18+ years who identified as Black, Latinx, Indigenous, or Asian. Categorical variables were summarized by frequency and percentage. The chi-square test was used to assess the relationship between responses and demographic variables. Statistical significance was based on *P*<.05. No multiple testing adjustment was performed. All analysis was done by R (version 4.0.5; R Foundation for Statistical Computing).

### Ethical Considerations

This study was exempt from approval by the University of California, San Francisco Institutional Review Board (IRB #22-36156). Informed consent was collected. All data collected were anonymized. Participants were not compensated.

## Results

Overall, 230 participants enrolled, and 199 surveys were completed (Table 1). While familiarity with AI was high, a smaller proportion of participants understood how AI can be used in health care (152/199, 76.4%), and an even smaller proportion understood how AI can be applied to dermatology (133/199, 66.8%). These outcomes did not vary significantly when stratifying by demographics such as race, age, gender, income, insurance type, or schooling (all *P*>.05).

**Figure 1:**
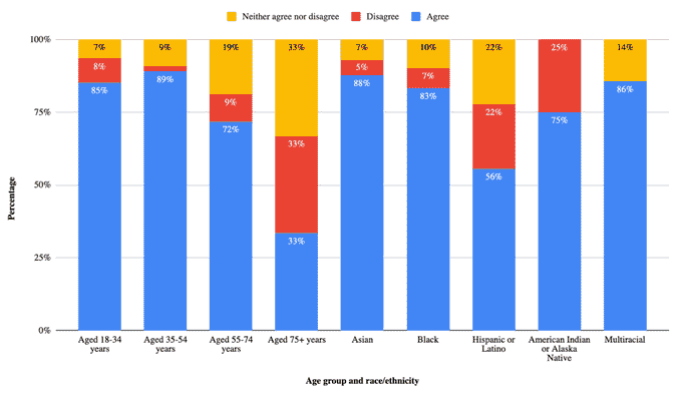
Percentage of participants who would like to be compensated if their medical information was being used for AI by age group and race/ethnicity.

Most participants (139/199, 69.8%) agreed that they trusted the health care system to treat their medical information with respect, which varied significantly by income (*P*=.045). Patients with lower incomes often experience more structural barriers to health care, and this could be one factor contributing to our finding that they are not as likely to trust the health care system. Only 64.3% (128/199) participants felt comfortable with their medical information being used to build AI tools. Most (181/199, 91%) want to be notified if their medical information is used in such a way. This varied by race (*P*=.002) and age (*P*=.03). Most (166/199, 83.4%) agreed they should be compensated if their medical information is used to develop AI, which varied by age (*P*=.045; Figure 1).

**Table 1:** Demographics of survey participants.

Demographics			Value (n=199), n (%)
**Gender**			
	Female	131 (65.8)	
	Male	63 (31.7)	
	Other or unknown	5 (2.5)	
**Age group (years)**			
	18-34	108 (54.3)	
	35-54	55 (27.6)	
	55-74	32 (16.1)	
	75+	3 (1.5)	
	Other or unknown	1 (0.5)	
**Race and ethnicity**			
	American Indian or Alaskan Native	4 (2)	
	Asian	57 (28.6)	
	Black	102 (51.3)	
	Hispanic or Latino	9 (4.5)	
	Multiracial	21 (10.6)	
	White	4 (2)	
	Other or unknown	2 (1)	
**Schooling**			
	High school or lower	19 (9.5)	
	Associate’s degree or trade school	30 (15.1)	
	Bachelor’s degree	85 (42.7)	
	Graduate degree	62 (31.2)	
	Other or unknown	3 (1.5)	
**Insurance**			
	Private insurance	109 (54.8)	
	Medicare or Medicaid	62 (31.2)	
	TRICARE or veterans	7 (3.5)	
	Indian Health Service	2 (1)	
	No health insurance	6 (3)	
	Other or unknown	13 (6.5)	
**Household income (US $)**			
	Less than 25,000	33 (16.6)	
	25,000-50,000	44 (22.1)	
	50,000-100,000	53 (26.6)	
	100,000-250,000	46 (23.1)	
	More than 250,000	12 (6)	
	Other or unknown	11 (5.5)	
**Geography**			
	Suburban	80 (40.2)	
	Urban	93 (46.7)	
	Rural	21 (10.6)	
	Other or unknown	5 (2.5)	
**Number of visits to the doctor in a year**			
	0-2	64 (32.2)	
	3-5	71 (35.7)	
	6+	61 (30.7)	
	Other or unknown	3 (1.5)	

## Discussion

To our knowledge, this is the first study focused on understanding opinions about health data use for AI among people who identify as Black, Latinx, Indigenous, or Asian. Similar studies have had limited diversity [4,5]. It is important to capture the opinions of diverse groups because the inclusion of their data is essential for building equitable AI tools; however, historical harms have made robust inclusion challenging. For racial and ethnic minority communities, historical experiences of racism, discrimination, and exploitation (eg, medical experimentation on populations without informed consent) may contribute to distrust in the health care system and, hence, decrease their comfort level with their data being used to develop AI systems. This suggests that AI development may not be inclusive, ethical, or aligned with the concerns of racial and ethnic minority communities enough to be safely deployed in health care settings. Ignoring these concerns could deepen distrust in AI, leading to lower rates of research participation in these already underrepresented communities, deepening biases and inequities in industries where AI is being deployed.

The educational gaps in AI uncovered by this inquiry should be addressed, as a fundamental understanding of AI is essential for patients to offer fully informed consent for health data use in AI development. There are existing initiatives addressing these educational gaps [6].

Most participants agreed that they should be notified if their data are used to build AI tools, which is not the current convention. For example, in dermatology, photos taken for clinical monitoring can be repurposed as data in AI tools without patient permission as long as they are “deidentified.”

Over 80% of participants agreed that they should be compensated when their data are used to build AI tools. Community-based participatory research shares findings and benefits with study participants. Sharing revenue with patients who contribute their data to build these valuable tools is thematically similar to sharing benefits of a work product and is something that should be explored.

Limitations include the study population. Participants were recruited from an academic medical center and research database, meaning that they may not represent the broader population. Future research should include a more diverse and representative sample from various backgrounds and regions to enhance generalizability.

## Abbreviations

AI: artificial intelligence

## References

[1] Omiye JA, Lester JC, Spichak S, Rotemberg V, Daneshjou R (2023). Large language models propagate race-based medicine. NPJ Digit Med.

[2] Daneshjou R, Vodrahalli K, Novoa RA, Jenkins M, Liang W, Rotemberg V, Ko J, Swetter Susan M, Bailey Elizabeth E, Gevaert Olivier, Mukherjee Pritam, Phung Michelle, Yekrang Kiana, Fong Bradley, Sahasrabudhe Rachna, Allerup Johan A C, Okata-Karigane Utako, Zou James, Chiou Albert S (2022). Disparities in dermatology AI performance on a diverse, curated clinical image set. Sci Adv.

[3] Adamson A, Smith A (2018). Machine learning and health care disparities in dermatology. JAMA Dermatol.

[4] Nelson CA, Pérez-Chada LM, Creadore A, Li SJ, Lo K, Manjaly P, Pournamdari AB, Tkachenko E, Barbieri JS, Ko JM, Menon AV, Hartman RI, Mostaghimi A (2020). Patient perspectives on the use of artificial intelligence for skin cancer screening: a qualitative study. JAMA Dermatol.

[5] Salvador T, Gu L, Hay JL, Kurtansky NR, Masterson-Creber R, Halpern AC, Rotemberg V (2024). Consent and identifiability for patient images in research, education, and image-based artificial intelligence. JAMA Dermatol.

[6] AI4ALL.

